# Injured proximal tubule-driven paracrine signaling promotes lipid-associated macrophage expansion and chondroitin sulfate biosynthesis via PPARG/TCF12 in diabetic kidney disease

**DOI:** 10.3389/fimmu.2026.1882611

**Published:** 2026-08-20

**Authors:** Bingshu Wu, Zihan Dou, Ming Cai, Siying Fei, Suxuan Liu, Xiaobin Mei

**Affiliations:** 1 Department of Geriatrics, The First Affiliated Hospital of Naval Medical University, Shanghai, China; 2 Department of Nephrology, The First Affiliated Hospital of Naval Medical University, Shanghai, China; 3 School of Pharmaceutical Sciences and Shanghai Engineering Research Center of Immunotherapeutics, Fudan University, Shanghai, China; 4 School of Basic Medical Sciences, Naval Medical University, Shanghai, China

**Keywords:** chondroitin sulfate, diabetic kidney disease, lipid-associated macrophage, PPARG, single-cell transcriptomics, TCF12

## Abstract

**Background:**

Diabetic kidney disease (DKD) is a leading cause of end-stage renal disease, yet its immune-mediated pathogenesis remains incompletely understood. Lipid-associated macrophages (LAMs) have emerged as key mediators of metabolic tissue injury, but their role in DKD and the upstream signals governing their transcriptional programs are undefined.

**Methods:**

We performed integrative single-cell transcriptomic analysis of 133,303 kidney cells (GSE211785), incorporating pseudotime trajectory analysis, SCENIC regulon inference, and CellChat/NicheNet intercellular communication modeling. Key findings were validated in an independent bulk RNA-seq cohort (GSE142025, n = 36) and *in vitro* by stimulating CD14^+^ monocyte-derived macrophages with conditioned medium from iPT-like HK-2 cells, followed by siRNA knockdown of PPARG, TCF12, and ANXA2.

**Results:**

LAMs were markedly expanded in DKD kidneys (~6.2-fold; 13.1% vs. 2.1% in controls) across both SC-RNA-seq and SN-RNA-seq platforms. SCENIC identified PPARG and TCF12 as dominant LAM-enriched transcription factor regulons, with downstream target genes converging on chondroitin sulfate (CS) biosynthesis via CHST11 and CSGALNACT1. CellChat revealed that iPT exhibited the highest incoming interaction strength toward LAM among all analyzed cell populations in DKD. NicheNet prioritized 11 candidate iPT-derived ligands preferentially targeting PPARG and TCF12, nine of which were independently elevated in advanced diabetic nephropathy. *In vitro*, iPT conditioned medium upregulated PPARG, TCF12, CHST11, CSGALNACT1, and intracellular CS in macrophages. siRNA knockdown of ANXA2 in HK-2 cells significantly attenuated CM-induced PPARG and TCF12 upregulation in macrophages, identifying ANXA2 as a key paracrine mediator.

**Conclusions:**

We define a novel iPT-to-LAM paracrine axis in DKD wherein tubular injury signals drive macrophage transcriptional reprogramming through PPARG and TCF12, culminating in aberrant CS biosynthesis. These findings implicate LAM-associated glycosaminoglycan remodeling as a previously unrecognized mechanism in DKD pathogenesis and identify PPARG/TCF12 as candidate therapeutic targets.

## Introduction

Diabetic kidney disease (DKD) is the most prevalent microvascular complication of diabetes mellitus and the leading cause of end-stage renal disease (ESRD) worldwide (1). Despite intensive glycemic and blood pressure control, a substantial proportion of patients progress to ESRD, highlighting the need for mechanistic insights that extend beyond metabolic perturbations (2). Chronic innate immune activation is increasingly recognized as a critical driver of DKD progression, and macrophage infiltration is a consistent histological feature across disease stages (3, 4).

Macrophages exhibit remarkable transcriptional plasticity, adopting context-specific phenotypes in response to local microenvironmental cues (5). Among recently characterized macrophage subsets, lipid-associated macrophages (LAMs) have attracted growing interest. First identified in adipose tissue and atherosclerotic plaques, LAMs are defined by high expression of lipid metabolism regulators — including PPARG, ABCG1, and MITF — and have been linked to TREM2-dependent lipid handling (6, 7). LAMs are now recognized across multiple metabolic disease contexts, including non-alcoholic steatohepatitis, obesity, and atherosclerosis, where they occupy functionally ambivalent roles by simultaneously attempting lipid clearance and sustaining local inflammation (8, 9). Whether a comparable LAM population expands in the DKD kidney, what upstream signals govern its emergence, and what downstream effector programs it executes have not been investigated.

The proximal tubule is among the most metabolically active nephron segments and one of the first to sustain injury in the diabetic milieu. Under hyperglycemic and profibrotic conditions, proximal tubular cells undergo a transcriptional transition to an injured state (iPT), marked by loss of differentiation markers, acquisition of an inflammatory secretome, and upregulation of damage-associated molecules including VCAM1, HAVCR1, and SPP1 (10, 11). iPT cells adopt a senescence-associated secretory phenotype (SASP) and have been shown to actively communicate with surrounding immune cells, including macrophages, to promote inflammation and fibrosis (12–14). However, the specific signals by which injured tubular epithelium instructs macrophage transcriptional reprogramming toward a LAM state in DKD have not been systematically defined.

Chondroitin sulfate (CS) is a sulfated glycosaminoglycan (GAG) and a structural component of the extracellular matrix. CS and related proteoglycans are known to accumulate in fibrotic renal diseases including diabetic nephropathy, interstitial fibrosis, and mesangial sclerosis, where CS deposition contributes to ECM stiffening and a pro-fibrotic microenvironment (15, 16). CS biosynthesis proceeds through a coordinated enzymatic cascade involving N-acetylgalactosaminyltransferases — notably CSGALNACT1 — and sulfotransferases, including CHST11, which catalyzes 4-O sulfation of CS chains and is upregulated in fibrotic conditions (17, 18). Despite this established role in renal fibrosis, the regulation of CS biosynthesis within kidney macrophages in DKD and its pathophysiological relevance remain unexplored.

In the present study, we integrated large-scale single-cell transcriptomics, pseudotime trajectory analysis, transcription factor regulon inference (SCENIC), and intercellular communication modeling (CellChat/NicheNet) with *in vitro* mechanistic experiments to define a novel iPT-to-LAM paracrine axis in DKD. Our findings reveal a previously unrecognized link between tubular injury and macrophage-mediated GAG remodeling, with implications for understanding the immune-stromal crosstalk that drives DKD progression.

## Materials and methods

### Single-cell RNA sequencing data acquisition and quality control

Raw single-cell and single-nucleus RNA sequencing (scRNA-seq/snRNA-seq) data were obtained from the Gene Expression Omnibus (GEO; accession number GSE211785), as previously described by Zhou et al. (19). This dataset comprises kidney biopsy samples from 20 healthy controls and 10 DKD patients profiled by both single-cell (SC, n = 54,416 cells) and single-nucleus (SN, n = 78,887 cells) platforms, yielding 133,303 cells in total after quality control (87,574 control cells; 45,729 DKD cells).

Raw count matrices were processed using Seurat (v5.2.1) (20). Quality control thresholds were as follows: genes detected per cell between 200 and 6,000; mitochondrial gene fraction below 25% for SC-RNA-seq and below 5% for SN-RNA-seq. All deposited cells passed these thresholds and were retained. Data were log-normalized (scale factor = 10,000), and the top 2,000 highly variable genes were identified. After scaling, data were subjected to principal component analysis (PCA; top 50 PCs). Platform-associated batch effects between SC and SN data were corrected using Harmony (21). Corrected embeddings were used for shared nearest-neighbor (SNN) graph construction and Leiden clustering (resolution = 0.5). UMAP was used for dimensionality reduction and visualization. Cell type annotations encompassing 41 distinct populations were transferred from the original Zhou et al. study.

### Myeloid subtype identification and characterization

Myeloid cells annotated as Mac, CD14_Mono, and CD16_Mono were extracted, yielding 5,964 cells. The Harmony-corrected embedding (top 30 dimensions) was used directly for SNN graph construction and Leiden clustering (resolution = 0.6). Two low-quality clusters were removed: cluster 3 (mean mitochondrial fraction = 4.71%; mean nFeature = 665) and cluster 7 (top markers predominantly mitochondrial). A myeloid-specific UMAP was generated (min.dist = 0.3, spread = 1). Five subtypes were annotated using canonical markers and differentially expressed genes (FindAllMarkers; Wilcoxon rank-sum test, min.pct = 0.25, log2FC threshold = 0.5): Resident_Mac (C1QA, C1QB, C1QC, FOLR2, MRC1, CD163), LAM (PPARG, ABCG1, MITF, KCNMA1, TMEM163, BNC2), NC_Mono (FCGR3A, CX3CR1, LST1, CDKN1C), IFN_Mono (ISG15, OAS1, STAT1), and Inflammatory_Mono (S100A8, S100A9, VCAN, IL1B, CXCL8, FCN1).

### Pseudotime trajectory analysis

Pseudotime trajectory analysis was performed on all 5,964 myeloid cells using Monocle3 (v1.4.26) (22). The myeloid UMAP embedding from Seurat was imported directly into the CellDataSet (CDS) object to preserve trajectory-cluster correspondence. A single partition was assigned to enforce a globally connected trajectory. The principal graph was learned using learn_graph (minimal_branch_len = 15, nn.k = 20, ncenter = 250), and cells were ordered via order_cells with automatic root selection. Genes varying systematically along the trajectory were identified by Moran’s I spatial autocorrelation test (graph_test; neighbor_graph = “principal_graph”, cores = 4). Genes meeting the thresholds q < 0.05 and Moran’s I > 0.2 were retained after excluding ribosomal (RPL/RPS), mitochondrial (MT-), and housekeeping genes (MALAT1, ACTB, GAPDH, B2M, TMSB4X, FTH1), yielding 200 trajectory-associated genes.

### Single-cell regulatory network inference

Transcription factor (TF) regulon activity was inferred using pySCENIC (v0.12) (23) following the standard three-step pipeline. Up to 500 cells per subpopulation were randomly sampled, and the top 3,000 highly variable genes (by Seurat variance-stabilizing transformation) were retained, supplemented by known marker and candidate TF genes. First, gene regulatory networks were inferred using GRNBoost2 with the human TF list from AnimalTFDB (v3.0) as candidate regulators (–num_workers 6, –seed 42). Second, putative regulons were pruned using the cisTarget algorithm against the hg38 ranking database (hg38:refseq-r80:10kb_up_and_down_tss.mc9nr), retaining only TF-target relationships with significant binding motif enrichment (motifs-v9-nr.hgnc-m0.001-o0.0). Third, regulon activity was scored per cell using AUCell, yielding an AUC matrix that was z-score normalized per subpopulation for visualization. Regulon activity differences between LAM and other subpopulations were assessed by Wilcoxon rank-sum test.

To identify LAM-enriched regulons with functional relevance to differentiation, we performed a three-way intersection among: (1) SCENIC-inferred TF regulons (n = 15); (2) SCENIC-predicted target genes (n = 451); and (3) pseudotime-associated Moran’s I genes (n = 200). This yielded 3 TF regulons — PPARG, TCF12, and FOXN3 — with 55 overlapping target genes (19 unique after deduplication), which were subjected to Reactome pathway enrichment analysis using ReactomePA (v1.44) with Benjamini-Hochberg correction.

### CellChat intercellular communication analysis

Intercellular communication between PT subpopulations and mononuclear phagocytes was analyzed using CellChat (v2.0) (24), run separately on Control and DKD condition cells. Signaling pathways were inferred from curated ligand-receptor interaction databases. Outgoing interaction strength per cell type was quantified using netAnalysis_computeCentrality (mean outdegree centrality across all pathways) and compared between conditions. To identify the dominant signaling source toward LAM, incoming interaction strength toward LAM from all analyzed cell populations was additionally computed from the CellChat weight matrix in DKD condition cells.

### NicheNet ligand activity prediction

NicheNet (v1.0) (25, 26) was applied to identify candidate iPT-derived ligands associated with LAM transcriptional reprogramming, using iPT as the sender and LAM as the receiver population (DKD cells only). The target gene set comprised the intersection of Moran’s I trajectory genes (n = 200) with the NicheNet ligand-target matrix (n = 194 after intersection). Candidate ligands were defined as iPT-expressed genes (pct > 0.10) whose cognate receptors were expressed in LAM. Ligand activity was scored using predict_ligand_activities (corrected AUPR), and significance was assessed by permutation test (n = 1,000; BH-adjusted p < 0.05). Significant ligands were further filtered by differential expression between iPT and PT_S1/S2/S3 (FindMarkers, log2FC > 2), yielding 11 final candidate ligands: ANXA2, ANXA1, THBS2, CCN1, VCAM1, VCAN, NAMPT, TNC, LAMC1, COL8A1, and CLDN1. The regulatory potential of these ligands toward 8 target genes (PPARG, TCF12, FOXN3, CHST11, CSGALNACT1, HS3ST2, ABR, TRIO) was extracted from the NicheNet ligand-target matrix, z-score normalized per target, and visualized as a heatmap. Receptor interactions for the 11 candidate ligands expressed in LAM were additionally extracted from the NicheNet ligand-receptor network to characterize downstream signaling axes.

### Bulk RNA-seq validation

Expression of iPT-derived candidate ligands was validated in the GSE142025 bulk RNA-seq dataset (27), comprising kidney biopsies from Control (n = 9), Early DN (n = 6), and Advanced DN (n = 21) subjects with TPM-normalized values. Comparisons between Control and Advanced DN groups were performed using the Mann-Whitney U test (two-tailed); p-values were not adjusted across ligands. Significance thresholds: *p < 0.05*, *p < 0.01*, p < 0.001, ****p < 0.0001.

### HK-2 cell culture, iPT-like model induction, and siANXA2 knockdown

HK-2 cells (ATCC CRL-2190) were maintained in high-glucose DMEM supplemented with 10% FBS and 1% penicillin-streptomycin at 37 °C in 5% CO_2_, and used within 30 passages. For iPT-like induction, cells grown to 70–80% confluence were synchronized by 24 h serum starvation in serum-free low-glucose DMEM (5.5 mM glucose) before treatment in four groups for 48 h: NC (5.5 mM D-glucose), MA (5.5 mM D-glucose + 24.5 mM D-mannitol; osmotic control), HG (30 mM D-glucose), and HG+TGF (30 mM D-glucose + TGF-β1–10 ng/mL). For ANXA2 knockdown experiments, HK-2 cells were transfected with siRNA targeting ANXA2 (siANXA2; MCE pre-designed siRNA) or negative control siRNA (siNC) using siRNA/miRNA Transfection Reagent (MCE, HY-K2051) for 48 h prior to iPT induction. Knockdown efficiency was confirmed by RT-qPCR. After 48 h induction, cells were washed twice with PBS and incubated for a further 24 h in fresh serum-free low-glucose DMEM. Conditioned medium (CM) from siNC-HG+TGF and siANXA2-HG+TGF cells was collected, cleared by centrifugation (300 × g, 10 min), and stored at −80 °C.

### CD14^+^ monocyte isolation, macrophage differentiation, and siRNA transfection

Cryopreserved human peripheral blood leukopaks were purchased from Hycells Biotechnology (Beijing, China; Catalog No. hPBLP001). Leukopaks were obtained from a healthy adult donor confirmed negative for HAV, HBV, HCV, HIV, HTLV, EBV, and B19. PBMCs were isolated by density gradient centrifugation according to standard protocols, and CD14^+^ monocytes were enriched by positive selection using anti-human CD14 magnetic beads (IPHASE Biosciences, Beijing, China) per manufacturer instructions. Monocytes were differentiated into macrophages by treatment with M-CSF (50 ng/mL) for 6 days, then rested in serum-free RPMI-1640 for 24 h. For knockdown experiments, cells were seeded at 3 × 10^5^ per well in 6-well plates and transfected with siRNA targeting PPARG or TCF12 (MCE pre-designed sets A) or negative control siRNA (siNC) using siRNA/miRNA Transfection Reagent (MCE, HY-K2051) for 48 h prior to CM stimulation.

### Conditioned medium stimulation and downstream analysis

Transfected macrophages were stimulated with 50% CM (diluted 1:1 with serum-free RPMI-1640) for 48 h. To assess paracrine specificity, an additional direct treatment group was included in which macrophages were exposed to HG+TGF conditions (30 mM D-glucose + TGF-β1–10 ng/mL) without CM, to exclude direct effects of high glucose and TGF-β1 on macrophage gene expression. The following treatment groups were established: NC (MA-CM, no siRNA), HG+TGF-CM, HG+TGF-CM/siNC, HG+TGF-CM/siPPARG, HG+TGF-CM/siTCF12, HG+TGF direct (no CM), and siANXA2-CM. Total RNA was extracted (Beyotime R0016) for RT-qPCR quantification of PPARG, TCF12, FOXN3, CHST11, and CSGALNACT1 (normalized to HPRT1). Protein lysates were prepared in IP lysis buffer, and total protein was quantified by BCA assay. Intracellular chondroitin sulfate was measured by competitive inhibition ELISA (Cloud-Clone Corp., UCEA723Ge) and expressed as pg/mg protein. Primer sequences are provided in **Supplementary Tables 1** and **S2**. Data represent mean ± SEM from n = 3 (RT-qPCR) or n = 5 (ELISA) independent biological replicates. Between-group comparisons used one-way ANOVA with Tukey’s *post hoc* test; p < 0.05 was considered statistically significant.

## Results

### Single-cell transcriptomic landscape of the human DKD kidney

**Figure 1:**
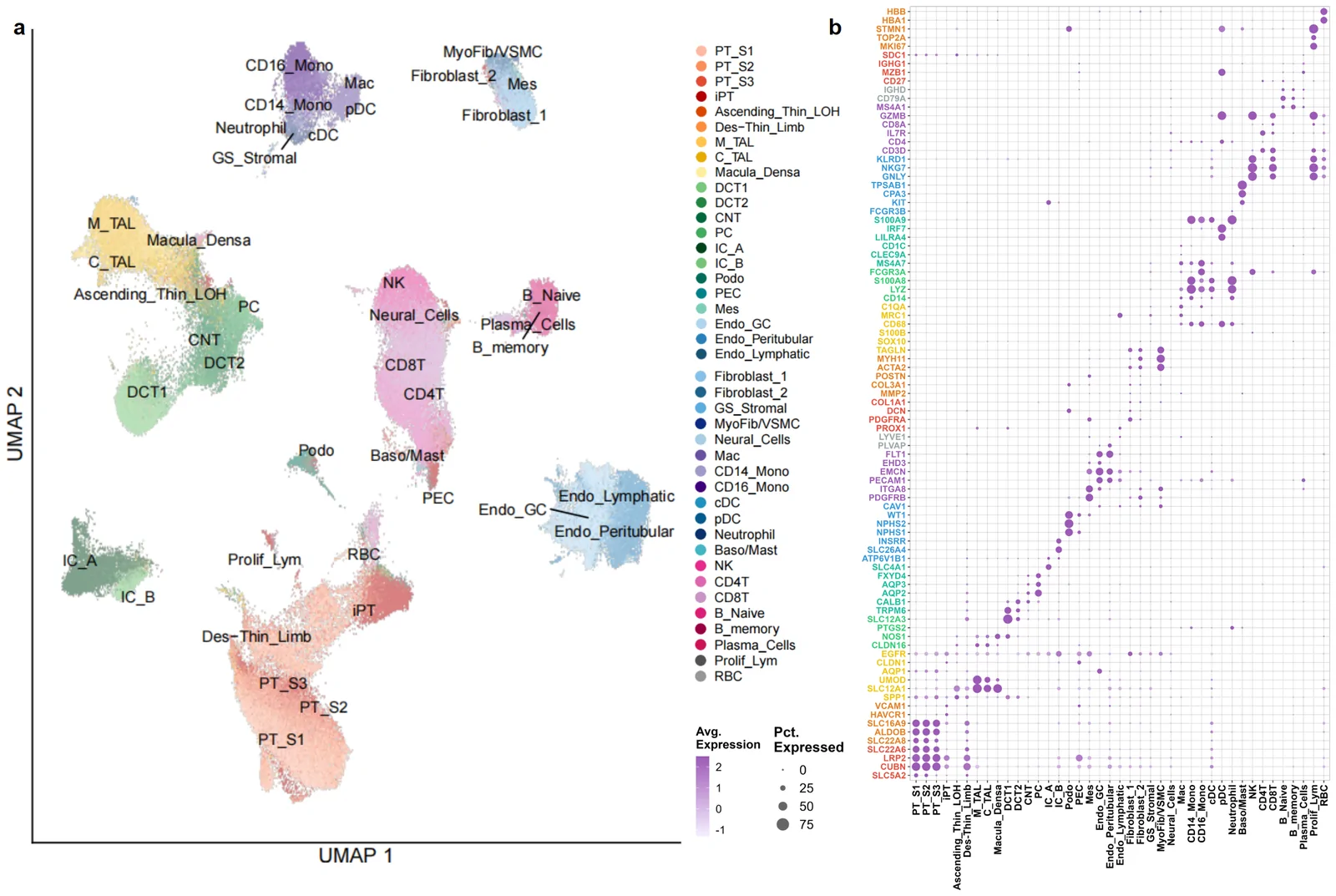
Single-cell transcriptomic landscape of the human DKD kidney. **(a)** UMAP visualization of 133,303 cells from 20 control and 10 DKD kidney biopsy samples, colored by annotated cell type. A total of 41 distinct populations were identified spanning major kidney compartments, including proximal tubule (PT_S1, PT_S2, PT_S3, and injured PT [iPT]), loop of Henle, distal tubule, collecting duct, glomerular, endothelial, stromal, myeloid, and lymphoid lineages **(b)** Dot plot showing the expression of canonical marker genes across all 41 cell types, including *SLC5A2*, *CUBN*, and *LRP2* for PT cells; *HAVCR1*, *VCAM1*, and *SPP1* for iPT; and *CD68*, *MRC1*, and *C1QA* for macrophages. Dot size indicates the percentage of cells expressing each gene; color intensity reflects the scaled average expression level.

To characterize the renal cellular landscape at single-cell resolution, we analyzed 133,303 kidney cells from 20 healthy controls and 10 DKD patients (GSE211785) using an integrated sc/snRNA-seq pipeline. Following Harmony-based batch correction between the SC (n = 54,416) and SN (n = 78,887) platforms, UMAP visualization identified 41 distinct cell populations spanning all major kidney compartments, including proximal tubule segments (PT_S1, PT_S2, PT_S3, and injured PT [iPT]), loop of Henle, distal tubule and collecting duct, glomerular, endothelial, stromal, and myeloid and lymphoid immune populations (**Figure 1a**). Cell type identity was confirmed by expression of compartment-specific marker genes, including *SLC5A2*, *CUBN*, and *LRP2* for PT cells; *HAVCR1*, *VCAM1*, and *SPP1* for iPT; and *CD68*, *MRC1*, and *C1QA* for macrophages (**Figure 1b**).

### Selective expansion of lipid-associated macrophages and impaired monocyte-to-macrophage differentiation in DKD

**Figure 2:**
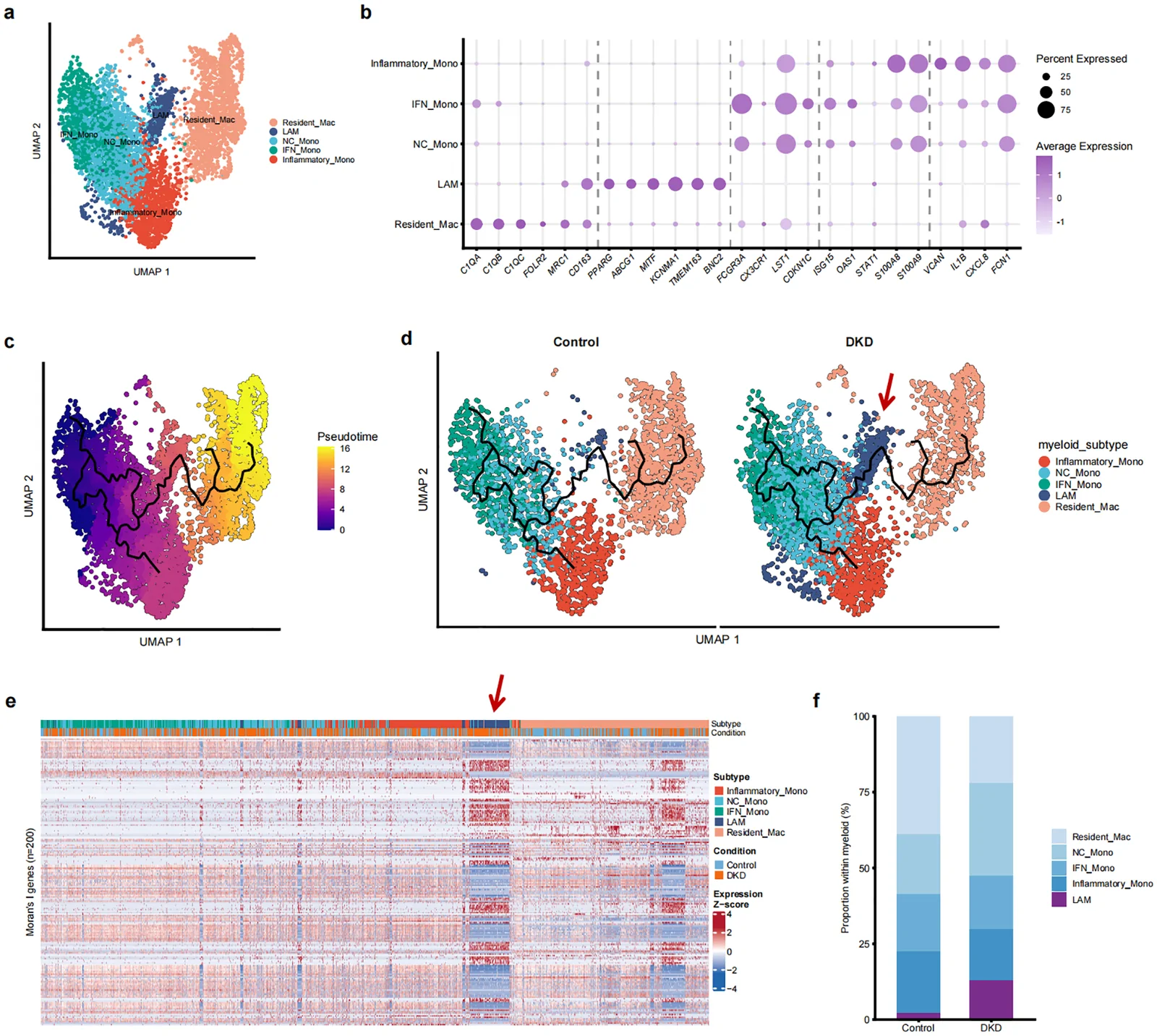
Selective expansion of lipid-associated macrophages and impaired monocyte-to-macrophage differentiation in DKD. **(a)** UMAP of 5,964 myeloid cells colored by subtype: Resident_Mac, LAM, NC_Mono, IFN_Mono, and Inflammatory_Mono **(b)** Dot plot of canonical marker gene expression across the five myeloid subtypes. Dot size indicates percentage of expressing cells; color intensity reflects scaled average expression. Dashed lines demarcate marker gene groups per subtype **(c)** Pseudotime trajectory of myeloid cells (Monocle3), with cells colored by pseudotime value (plasma scale) and the principal graph overlaid **(d)** Split UMAP showing pseudotime trajectory in Control and DKD conditions. Red arrow indicates preferential accumulation of DKD cells at the LAM terminal branch **(e)** Heatmap of Moran’s I-significant genes (n = 200; q < 0.05, Moran’s I > 0.2) ordered by pseudotime peak expression. Annotation bars indicate cell subtype (upper) and condition (lower). Color scale: expression Z-score (capped at ±2.5). Red arrow indicates the LAM-enriched expression module **(f)** Stacked bar chart of myeloid subtype proportions in Control and DKD. LAM expanded from 2.1% (56/2,629 cells) in controls to 13.1% (436/3,335 cells) in DKD, representing an approximately 6.2-fold increase.

Unsupervised subclustering of 5,964 myeloid cells resolved five transcriptionally distinct subpopulations: Resident macrophages (Resident_Mac), lipid-associated macrophages (LAM), non-classical monocytes (NC_Mono), IFN-stimulated monocytes (IFN_Mono), and inflammatory monocytes (Inflammatory_Mono) (**Figure 2a**). Each subtype displayed a characteristic marker gene signature (**Figure 2b**). The LAM cluster was distinguished by high expression of the lipid metabolism transcription factor PPARG (detection rate 40.2%), cholesterol transporter ABCG1 (37.0%), and transcription factor MITF (45.9%), consistent with the canonical LAM signature (6).

LAMs were substantially expanded in DKD relative to controls (436/3,335 cells, 13.1% vs. 56/2,629 cells, 2.1%; approximately 6.2-fold increase; **Figure 2f**). To confirm that this expansion was not driven by outlier samples, we examined LAM frequencies across sequencing platforms: in SC-RNA-seq, LAM frequency increased from 0.04% in controls to 0.40% in DKD; in SN-RNA-seq, from 27.1% to 50.8%. The consistent directional expansion across both platforms (**Supplementary Figure 1**) supports a robust DKD-associated LAM enrichment rather than a platform-specific or sample-specific artifact.

Pseudotime trajectory analysis using Monocle3 positioned inflammatory and classical monocytes at the trajectory root, with Resident_Mac and LAM occupying separate terminal branches (**Figure 2c**). LAM accumulation at the terminal end of the trajectory was markedly greater in DKD than in control samples (**Figure 2d**, red arrow), indicating that the DKD microenvironment selectively promotes monocyte-to-LAM differentiation. Moran’s I analysis (q < 0.05, Moran’s I > 0.2) identified 200 genes varying systematically along this trajectory, capturing the transcriptional dynamics of LAM specification (**Figure 2e**).

### SCENIC identifies PPARG and TCF12 as the principal LAM transcription factor regulons, converging on chondroitin sulfate biosynthesis

**Figure 3:**
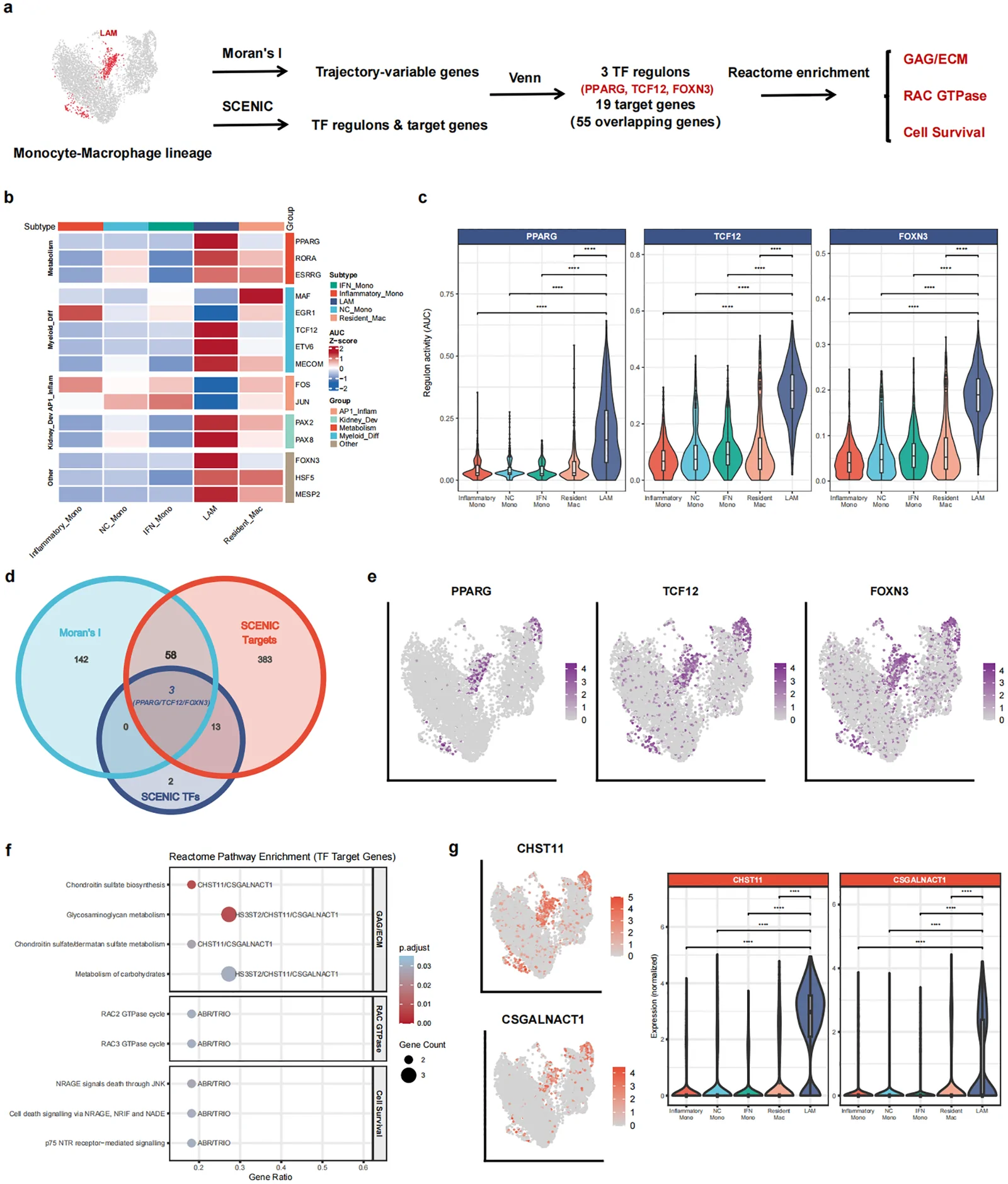
SCENIC-based identification of LAM-specific transcription factor regulons and their downstream target network. **(a)** Analytical workflow schematic. Moran’s I trajectory-variable genes (n = 200) and SCENIC-derived TF regulon target genes were integrated by Venn diagram analysis, yielding 3 TF regulons (PPARG, TCF12, FOXN3) and 19 unique target genes. Reactome enrichment identified three functional categories: GAG/ECM remodeling, RAC GTPase signaling, and cell survival **(b)** Heatmap of AUC z-scores for 15 SCENIC regulons across five myeloid subpopulations, grouped by functional category **(c)** Violin plots of PPARG, TCF12, and FOXN3 regulon activity (AUC) across subpopulations. LAM shows significantly higher activity than all other subpopulations (****p < 2.22×10^-^¹^6^, Wilcoxon rank-sum test) **(d)** Venn diagram of Moran’s I genes, SCENIC target genes, and SCENIC TF regulons. Triple intersection identifies PPARG, TCF12, and FOXN3 as LAM-enriched regulons with trajectory-variable target genes **(e)** Feature plots of PPARG, TCF12, and FOXN3 expression on the myeloid UMAP, showing enrichment in the LAM cluster **(f)** Reactome pathway enrichment of the 19 TF target genes. Pathways are grouped into GAG/ECM remodeling (driven by CHST11, CSGALNACT1, HS3ST2), RAC GTPase signaling, and cell survival. Dot size: gene count; color: adjusted p-value. Of the 19 target genes, 5 were significantly enriched in Reactome pathways (p.adjust < 0.05): CHST11, CSGALNACT1, and HS3ST2 (GAG/ECM), and ABR and TRIO (RAC GTPase/cell survival). The remaining 14 target genes (ATXN1, BNC2, CAMK1D, DISC1, ELMO1, EPB41L2, FAM53B, FMN1, KCNMA1, MGAT4A, PTPRJ, SFMBT2, SLCO2B1, SNTB1) did not reach significance in Reactome pathway enrichment and are provided in **Supplementary Table 3**. **(g)** Feature plots (left) and violin plots (right) of CHST11 and CSGALNACT1 across myeloid subpopulations, confirming selective enrichment in LAM (****p < 2.22×10^-^¹^6^, Wilcoxon rank-sum test).

To resolve the transcriptional regulatory architecture of the LAM state, we applied pySCENIC across the five myeloid subpopulations. Among 15 inferred regulons, those in the metabolism and myeloid differentiation categories showed the greatest LAM-specific enrichment (**Figure 3b**). PPARG, TCF12, and FOXN3 regulons each exhibited significantly higher activity in LAM than in all other subpopulations (p < 2.22×10^-^¹^6^, Wilcoxon rank-sum test; **Figure 3c**), and their expression was selectively enriched in the LAM region of the UMAP embedding (**Figure 3e**).

To link regulon activity to differentiation dynamics, we intersected SCENIC-inferred regulons (n = 15), SCENIC-predicted target genes (n = 451), and pseudotime-associated Moran’s I genes (n = 200) (**Figures 3a, d**). This three-way intersection identified PPARG, TCF12, and FOXN3 as LAM-enriched regulons whose target genes are dynamically regulated along the monocyte-to-LAM trajectory, yielding 19 unique downstream effectors. Notably, FOXN3 was predicted to be a direct downstream target of TCF12 by cisTarget motif enrichment, implying a sequential regulatory cascade in which TCF12 amplifies transcriptional output through FOXN3.

Reactome enrichment of the 19 target genes revealed three functional categories: GAG/ECM remodeling (chondroitin sulfate biosynthesis and glycosaminoglycan metabolism), RAC GTPase signaling, and cell survival (**Figure 3f**). The CS biosynthesis category was driven by CHST11, CSGALNACT1, and HS3ST2. Of the 19 target genes, 5 were significantly enriched in Reactome pathways (p.adjust < 0.05): CHST11, CSGALNACT1, and HS3ST2 (GAG/ECM remodeling), and ABR and TRIO (RAC GTPase signaling/cell survival). The remaining 14 target genes (ATXN1, BNC2, CAMK1D, DISC1, ELMO1, EPB41L2, FAM53B, FMN1, KCNMA1, MGAT4A, PTPRJ, SFMBT2, SLCO2B1, SNTB1) did not reach significance in Reactome pathway enrichment analysis and are provided in **Supplementary Table 3**. Both CHST11 and CSGALNACT1 were selectively enriched in LAM by UMAP projection and violin plot analysis (**Figure 3g**), confirming LAM-specific activation of this biosynthetic program downstream of PPARG and TCF12. Differential expression analysis of our single-cell dataset further revealed that macrophages exhibited the most significant upregulation of both CHST11 and CSGALNACT1 in DKD compared to control (CHST11: log2FC = 3.09, p = 4.85×10^-^¹¹²; CSGALNACT1: log2FC = 2.83, p = 8.47×10^-59^; **Supplementary Figure 2**), suggesting that DKD-associated macrophages, particularly LAM, represent a major cell population driving CS biosynthesis upregulation in the diabetic kidney microenvironment.

### iPT cells are the dominant paracrine signaling source toward LAMs in DKD, providing candidate ligands that target PPARG/TCF12

**Figure 4:**
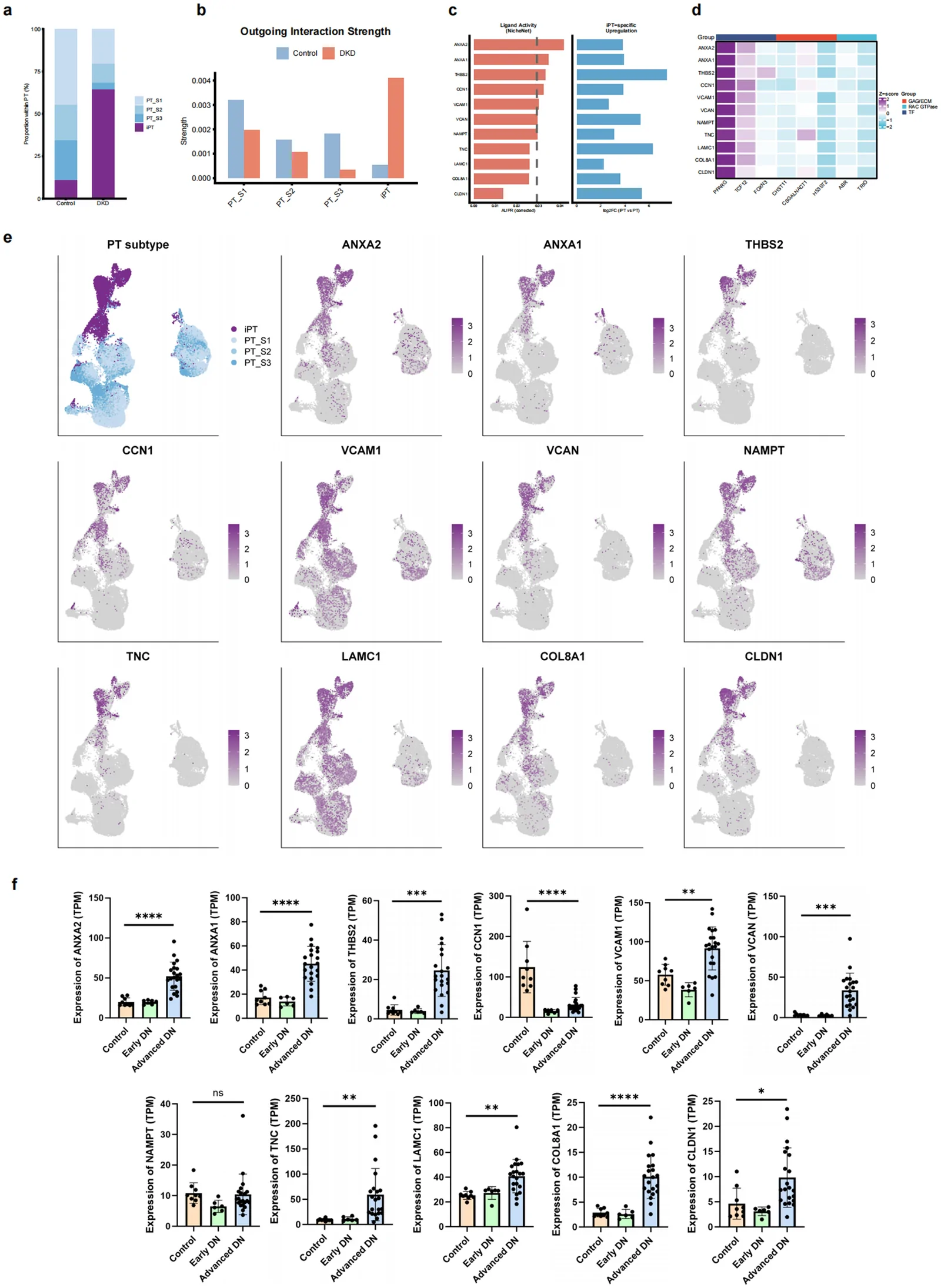
iPT-driven paracrine signaling reprograms LAM transcription through PPARG and TCF12, and is elevated in advanced diabetic nephropathy. **(a)** Stacked bar plot of PT subpopulation proportions (PT_S1, PT_S2, PT_S3, iPT) in Control and DKD. iPT expands markedly in DKD **(b)** Grouped bar plot of CellChat-inferred outgoing interaction strength for PT subpopulations in Control and DKD. iPT selectively increases outgoing communication in DKD; other PT subtypes show stable or reduced strength **(c)** Dual bar plot of NicheNet ligand activity (corrected AUPR, left) and iPT-specific upregulation (log2FC vs. PT_S1/S2/S3, right) for 11 candidate ligands **(d)** Heatmap of z-score normalized regulatory potential of 11 candidate ligands toward 8 target genes (TF regulons: PPARG, TCF12, FOXN3; effector genes: CHST11, CSGALNACT1, HS3ST2, ABR, TRIO). PPARG and TCF12 show consistently high regulatory potential. Analysis of the NicheNet ligand-receptor network identified TLR2 (40.4% of LAM cells), ITGAV (30.3%), CD44 (30.3%), and ITGB1 (16.3%) as the major receptors expressed in LAM, providing a mechanistic basis for how extracellular iPT-derived ligands activate intracellular PPARG/TCF12 signaling cascades **(e)** Feature plots of PT subtype annotation and 11 candidate ligands on the PT UMAP, confirming iPT-enriched expression **(f)** Bar plots of candidate ligand expression (TPM) in Control (n = 9), Early DN (n = 6), and Advanced DN (n = 21) kidney biopsies (GSE142025; Mann-Whitney U test, Control vs. Advanced DN). *p < 0.05*, *p < 0.01*, p < 0.001, ****p < 0.0001; ns, not significant.

Given the LAM expansion in DKD and the established role of the injured proximal tubule in modulating the renal immune microenvironment, we asked whether iPT cells supply the paracrine cues that drive LAM reprogramming. CellChat analysis revealed a marked and selective increase in iPT outgoing communication strength in DKD, whereas PT_S1, PT_S2, and PT_S3 showed stable or reduced strength (**Figures 4a, b**). Critically, analysis of incoming interaction strength toward LAM across all analyzed cell populations demonstrated that iPT exhibited the highest incoming interaction strength toward LAM in DKD (iPT: 1.45×10^-^³), exceeding PT_S1 by approximately 5-fold (2.94×10^-4^) and all monocyte subpopulations by more than 10-fold (IFN_Mono: 1.04×10^-4^; NC_Mono: 6.41×10^-5^; Inflammatory_Mono: 2.46×10^-5^), confirming iPT as the dominant signaling source toward LAM in DKD (**Supplementary Figure 3**). These findings are consistent with prior studies demonstrating that VCAM1^+^ injured proximal tubule cells adopt a SASP and actively communicate with surrounding immune cells to promote inflammation and fibrosis (12–14).

NicheNet analysis, with iPT as the sender and LAM as the receiver (DKD cells), identified 11 candidate ligands with significant activity (BH-adjusted p < 0.05) and iPT-specific upregulation (log2FC > 2 vs. PT_S1/S2/S3): ANXA2, ANXA1, THBS2, CCN1, VCAM1, VCAN, NAMPT, TNC, LAMC1, COL8A1, and CLDN1 (**Figure 4c**). Across all 11 candidates, PPARG and TCF12 consistently received the highest predicted regulatory input (**Figure 4d**), identifying these TFs as the principal transcriptional targets of iPT-derived signaling in LAMs. Feature plots confirmed enriched expression of all 11 ligands in the iPT cluster within the PT UMAP (**Figure 4e**).

Analysis of the NicheNet ligand-receptor network identified 84 receptor interactions for the 11 candidate ligands, with TLR2 (40.4% of LAM cells), ITGAV (30.3%), CD44 (30.3%), and ITGB1 (16.3%) among the most highly expressed receptors in LAM. These receptors link iPT-derived extracellular signals to intracellular PPARG/TCF12 activation through three major signaling axes: (1) TLR2/TLR4 → NF-κB/MAPK → PPARG, through which CCN1, ANXA2, and VCAN signal (28); (2) integrin (ITGAV/ITGB1) → FAK/PI3K/AKT → PPARG, through which TNC, LAMC1, COL8A1, THBS2, and VCAM1 activate PPARG transcriptional activity in macrophages (29); and (3) CD44 → PPARG lipid metabolism program, through which VCAN-CD44 signaling promotes M2-like macrophage polarization consistent with the LAM phenotype (30, 31). It should be noted that NicheNet provides a systems-level prioritization based on transcriptomic co-regulation; direct ligand-receptor causality requires future receptor-specific functional experiments.

To assess clinical relevance, we examined ligand expression in the independent GSE142025 bulk RNA-seq cohort (27). Nine of the 11 candidates — ANXA2, ANXA1, THBS2, VCAM1, VCAN, TNC, LAMC1, COL8A1, and CLDN1 — were significantly elevated in Advanced DN relative to controls (**Figure 4f**). NAMPT showed no significant change. CCN1 was paradoxically decreased in Advanced DN despite its upregulation in the HG+TGF iPT-like model. We attribute this discrepancy to progressive tubular atrophy in advanced disease: although individual injured tubular cells upregulate CCN1, the net loss of iPT mass at the tissue level results in a reduced bulk signal — a well-recognized limitation of bulk RNA-seq in progressive nephropathy.

### iPT conditioned medium activates PPARG/TCF12 and drives chondroitin sulfate biosynthesis in primary human macrophages via ANXA2-dependent paracrine signaling

**Figure 5:**
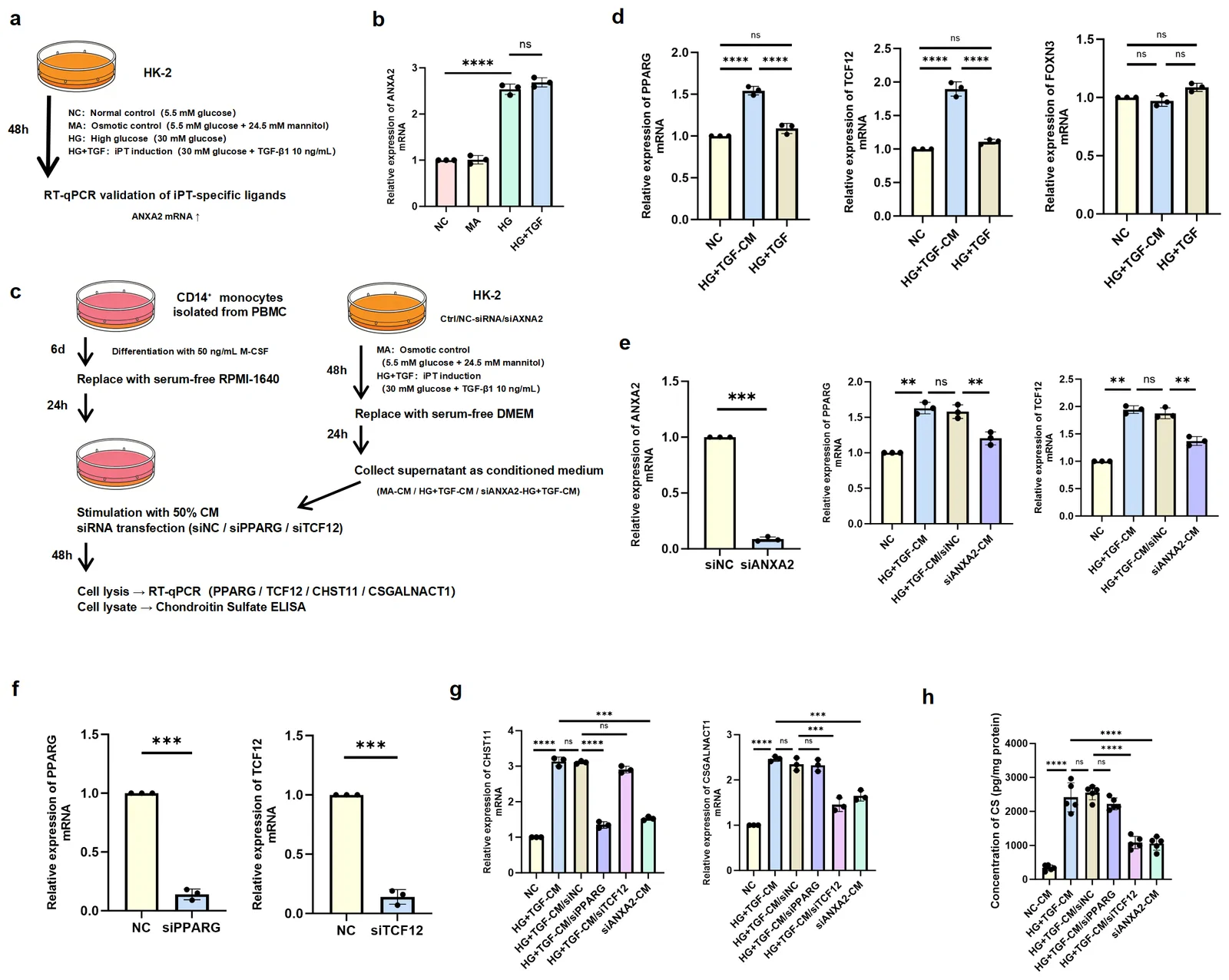
*In vitro* validation of the iPT-to-LAM communication axis and PPARG/TCF12-driven chondroitin sulfate biosynthesis. **(a)** Schematic of the iPT-like induction protocol in HK-2 cells. Treatment groups: NC (5.5 mM D-glucose), MA (5.5 mM D-glucose + 24.5 mM D-mannitol; osmotic control), HG (30 mM D-glucose), HG+TGF (30 mM D-glucose + TGF-β1–10 ng/mL). Panel A text indicates ANXA2 mRNA upregulation confirmed in HK-2 cells **(b)** RT-qPCR of ANXA2 mRNA expression in HK-2 cells after 48 h treatment, confirming iPT-like induction. Data normalized to ACTB; n = 3, mean ± SEM; one-way ANOVA with Tukey’s *post hoc* test; ****p < 0.0001; ns, not significant **(c)** Experimental workflow schematic. CD14^+^ monocytes were differentiated with M-CSF (6 days), rested (24 h), seeded at 3 × 10^5^ cells/well, transfected with siRNA (48 h), and stimulated with 50% CM (48 h). Readouts: RT-qPCR (PPARG, TCF12, FOXN3, CHST11, CSGALNACT1) and chondroitin sulfate ELISA **(d)** RT-qPCR of PPARG, TCF12, and FOXN3 in CD14^+^ monocyte-derived macrophages stimulated with MA-CM (NC), HG+TGF-CM, or direct HG+TGF treatment (without CM). PPARG and TCF12 are significantly upregulated by HG+TGF-CM but not by direct HG+TGF treatment, demonstrating that the effects are attributable to iPT-derived paracrine factors. FOXN3 is not significantly changed at this time point, consistent with its role as a delayed secondary TCF12 target. Data normalized to HPRT1; n = 3, mean ± SEM; one-way ANOVA with Tukey’s *post hoc* test; ****p < 0.0001; ns, not significant **(e)** Effect of ANXA2 knockdown in HK-2 cells on macrophage PPARG and TCF12 expression. Left: ANXA2 knockdown efficiency in HK-2 cells confirmed by RT-qPCR (siNC vs. siANXA2). Middle and right: PPARG and TCF12 mRNA expression in CD14^+^ monocyte-derived macrophages stimulated with NC, HG+TGF-CM, HG+TGF-CM/siNC, or siANXA2-CM. siANXA2-CM significantly attenuated HG+TGF-CM-induced upregulation of both PPARG and TCF12, identifying ANXA2 as a key paracrine mediator. Data normalized to HPRT1; n = 3, mean ± SEM; left panel: unpaired t-test; middle and right panels: one-way ANOVA with Tukey’s *post hoc* test; *p < 0.05*, *p < 0.01*, p < 0.001; ns, not significant **(f)** siRNA knockdown validation of PPARG (left) and TCF12 (right) in CD14^+^ monocyte-derived macrophages across treatment groups, confirming efficient and specific silencing without transfection-related off-target effects. Data normalized to HPRT1; n = 3, mean ± SEM; one-way ANOVA with Tukey’s; ****p < 0.0001; ns, not significant **(g)** RT-qPCR of CHST11 (left) and CSGALNACT1 (right) in CD14^+^ monocyte-derived macrophages following CM stimulation and siRNA knockdown across six groups: NC, HG+TGF-CM, HG+TGF-CM/siNC, HG+TGF-CM/siPPARG, HG+TGF-CM/siTCF12, and siANXA2-CM. siPPARG specifically reverses CHST11 upregulation; siTCF12 specifically reverses CSGALNACT1 upregulation, demonstrating reciprocal TF-target specificity concordant with SCENIC predictions. siANXA2-CM attenuated both CHST11 and CSGALNACT1, consistent with ANXA2 acting upstream of both transcription factors. Data normalized to HPRT1; n = 3, mean ± SEM; one-way ANOVA with Tukey’s; *p < 0.001*, *p < 0.0001; ns, not significant **(h)** Chondroitin sulfate concentrations in CD14^+^ monocyte-derived macrophage cell lysates (competitive ELISA, Cloud-Clone UCEA723Ge), normalized to total protein. CS is elevated by HG+TGF-CM stimulation; siTCF12 knockdown and siANXA2-CM stimulation each significantly reduced CS concentrations, consistent with the TCF12→CSGALNACT1→CS biosynthesis axis. siPPARG knockdown also significantly reduced CS levels, consistent with PPARG→CHST11 regulating CS sulfation. n = 5, mean ± SEM; one-way ANOVA with Tukey’s; ****p < 0.0001; ns, not significant.

To functionally validate the iPT-to-LAM axis, we established an *in vitro* model using CM from iPT-like HK-2 cells to stimulate CD14^+^ monocyte-derived macrophages (**Figures 5a, c**). Successful iPT-like induction was confirmed by RT-qPCR of ANXA2, which was significantly upregulated in HG and HG+TGF groups (**Figure 5b**). These expression patterns are consistent with the computational predictions and confirm model fidelity.

To exclude the possibility that high glucose and TGF-β1 directly affect macrophage gene expression independent of paracrine signals, we included a direct HG+TGF treatment group in which macrophages were exposed to 30 mM D-glucose + TGF-β1 (10 ng/mL) without CM. Direct HG+TGF treatment did not significantly induce PPARG, TCF12, CHST11, CSGALNACT1, or CS in macrophages (**Figure 5d**), demonstrating that the observed downstream effects are specifically attributable to iPT-derived paracrine factors rather than direct metabolic stimulation.

Macrophages stimulated with HG+TGF-CM showed significant upregulation of PPARG and TCF12 relative to MA-CM controls (**Figure 5d**). FOXN3 was not significantly induced at 48 h, consistent with its predicted role as a secondary TCF12 target whose induction likely requires sustained TF accumulation beyond the current stimulation window. Both CHST11 and CSGALNACT1 were significantly upregulated by HG+TGF-CM (**Figure 5g**), and intracellular CS concentrations were elevated at the metabolite level (**Figure 5h**), confirming activation of the full biosynthetic cascade.

To establish the causal contributions of PPARG and TCF12, we performed siRNA knockdown prior to CM stimulation. Knockdown efficiency was confirmed for both targets without off-target effects on the other TF (**Figure 5f**). siPPARG selectively reversed CHST11 upregulation without affecting CSGALNACT1; siTCF12 specifically reversed CSGALNACT1 upregulation without affecting CHST11 (**Figure 5g**). This reciprocal specificity is fully concordant with the SCENIC-predicted TF-target assignments. At the functional level, siTCF12 significantly reduced intracellular CS, and siPPARG also significantly reduced CS levels (**Figure 5h**), indicating that both PPARG→CHST11 and TCF12→CSGALNACT1 axes contribute to CS biosynthesis, with TCF12-driven CSGALNACT1 expression representing the primary determinant of CS chain elongation in this context.

To identify the key paracrine mediator responsible for CM-induced macrophage reprogramming, we performed siRNA-mediated knockdown of ANXA2 in HK-2 cells prior to iPT induction (**Figure 5e**). siANXA2 efficiently reduced ANXA2 mRNA expression in HK-2 cells, and CM derived from siANXA2-treated HK-2 cells (siANXA2-CM) significantly attenuated HG+TGF-CM-induced upregulation of both PPARG and TCF12 in CD14^+^ monocyte-derived macrophages (**Figure 5e**). This finding identifies ANXA2 as a key iPT-derived paracrine mediator of LAM transcriptional reprogramming and provides direct experimental support for the iPT → ANXA2 → PPARG/TCF12 signaling axis. Taken together, these experiments establish a mechanistic iPT → ANXA2 → PPARG/TCF12 → CHST11/CSGALNACT1 → CS signaling cascade in DKD-relevant macrophages.

## Discussion

This study delineates a novel intercellular signaling axis in DKD by which injured proximal tubular cells drive the expansion and transcriptional reprogramming of lipid-associated macrophages through PPARG- and TCF12-dependent activation of chondroitin sulfate biosynthesis. By integrating single-cell transcriptomics of a large, well-characterized human kidney cohort with *in vitro* mechanistic experiments, we identify LAMs as a markedly expanded myeloid population in DKD and define the regulatory logic and upstream paracrine cues that govern their differentiation and effector output.

Our finding of a ~6.2-fold LAM expansion in the DKD kidney extends an emerging paradigm from metabolic disease contexts to the diabetic renal microenvironment (8, 9). LAMs were first characterized in murine adipose tissue and atherosclerotic plaques, where TREM2-expressing macrophages accumulate in response to lipid overload (6, 7). Subsequent work identified LAMs in the liver and brain under conditions of elevated lipid burden and sterile inflammation (32, 33). In the kidney, ectopic lipid accumulation is a recognized feature of DKD (34), providing a plausible microenvironmental stimulus for LAM specification. Although TREM2 transcript detection was sparse in our dataset — likely attributable to scRNA-seq dropout — the canonical LAM signature encompassing PPARG, ABCG1, and MITF was robustly present, consistent with TREM2-independent LAM identification approaches used in recent studies (17). The consistent LAM expansion observed across both SC-RNA-seq and SN-RNA-seq platforms in our dataset further strengthens the robustness of this finding.

A central finding of this study is the mechanistic dissection of the transcriptional regulatory network that governs the LAM effector program. SCENIC identified PPARG as the principal lipid-metabolism regulon in LAMs — consistent with its established role as a master regulator of macrophage lipid handling and alternative activation (28, 35). We demonstrate that in the DKD renal context, PPARG activity is channeled toward chondroitin sulfate biosynthesis through direct regulation of CHST11, extending the known transcriptional targets of PPARG beyond the canonical lipid catabolism program.

TCF12, a class I basic helix-loop-helix transcription factor, has not previously been implicated in macrophage biology or kidney disease. We show that TCF12 is a LAM-enriched regulon that controls expression of CSGALNACT1 — the rate-limiting transferase that initiates chondroitin sulfate chain elongation — and, by cisTarget prediction, FOXN3. Our siRNA experiments provide the first functional evidence that TCF12 is required for CSGALNACT1 induction and CS accumulation in macrophages stimulated by tubular injury signals, establishing TCF12 as a novel regulator of GAG metabolism in innate immune cells. The TCF12 → FOXN3 cascade implied by motif enrichment analysis represents an additional layer of transcriptional amplification within the LAM program that merits further investigation. The absence of significant FOXN3 induction in our 48 h *in vitro* model is consistent with delayed induction kinetics expected for a secondary target; time-course experiments will be needed to resolve this cascade.

The upstream positioning of iPT cells as the dominant providers of LAM-activating paracrine signals is both mechanistically coherent and clinically relevant. The iPT state — defined by loss of tubular identity and acquisition of a SASP — has been characterized across multiple CKD and DKD datasets (10, 11). Injured proximal tubular cells have been shown to communicate with surrounding immune cells through secreted factors to promote inflammation and fibrosis (12–14). Our CellChat incoming interaction strength analysis confirms that iPT represents the strongest signaling sender toward LAM among all analyzed cell populations in DKD, exceeding all PT subtypes and monocyte populations. NicheNet analysis further identifies a set of 11 candidate ligands — spanning matrix-associated molecules (THBS2, TNC, LAMC1, COL8A1, VCAN), inflammatory mediators (VCAM1, ANXA1, ANXA2), and metabolic regulators (NAMPT) — predicted to activate the PPARG/TCF12 axis in LAMs through receptor-mediated signaling. Analysis of the NicheNet ligand-receptor network highlights TLR2, integrins (ITGAV/ITGB1), and CD44 as the major LAM-expressed receptors, linking iPT-derived extracellular signals to intracellular PPARG/TCF12 activation through TLR2/NF-κB/MAPK, integrin/FAK/PI3K/AKT, and CD44-mediated pathways (28–31). Our functional validation with siANXA2 knockdown in HK-2 cells identifies ANXA2 as a key paracrine mediator, providing direct experimental evidence for the iPT → ANXA2 → PPARG/TCF12 signaling axis. We acknowledge that bidirectional crosstalk between tubular epithelial cells and macrophages is well established in kidney disease (36), and that the reverse LAM→iPT axis may represent an equally important dimension of DKD pathology; future studies examining this reciprocal communication would be an important extension of this work.

The pathological consequences of macrophage-driven CS biosynthesis in the DKD kidney are likely to be pleiotropic. CS and related proteoglycans are known to accumulate in fibrotic renal diseases, where CS deposition contributes to ECM stiffening and a pro-fibrotic microenvironment (15, 16). CS chains shape the pericellular matrix, influencing growth factor bioavailability, inflammatory mediator presentation, and cell adhesion (17). Our single-cell data reveal that macrophages exhibit the most pronounced upregulation of CHST11 and CSGALNACT1 in DKD among all renal cell types, and our *in vitro* data establish that this upregulation is driven by iPT-derived paracrine signals through PPARG and TCF12. This points toward a non-canonical, GAG-mediated effector mechanism in DKD that operates in parallel with classical inflammatory cytokine secretion. We note that our current experimental system quantifies total intracellular CS and monitors transcript levels of biosynthetic enzymes, but does not directly resolve specific sulfation patterns. CHST11 catalyzes 4-O-sulfation of CS chains — a modification that critically determines CS biological activity and has been associated with fibrosis promotion through SMAD3 signaling (18) — and its precise substrate preference in DKD-associated macrophages remains to be characterized by LC-MS or sulfation-specific immunochemistry.

Several limitations warrant acknowledgment. First, our *in vitro* model relies on HK-2 cells and monocyte-derived macrophages as surrogates for iPT and LAM, respectively; confirmation in primary tubular epithelial cultures, kidney organoid co-culture systems, and *in vivo* DKD models will be important. Second, although ANXA2 was identified as a key paracrine mediator, the 11 candidate ligands likely act in combination, and receptor-specific causal links for the remaining ligands remain to be established. Third, the spatial localization of LAMs within the DKD kidney — whether peritubular, periglomerular, or interstitial — remains unresolved and could be addressed by spatial transcriptomics. Finally, whether PPARG/TCF12-driven CS accumulation in LAMs promotes or attenuates DKD progression, and whether targeting this pathway has therapeutic benefit, requires investigation in longitudinal clinical cohorts and preclinical models.

In summary, this study establishes the iPT-to-LAM paracrine signaling axis as a novel immune mechanism in DKD, in which tubular injury signals — particularly ANXA2 — converge on macrophage PPARG and TCF12 to activate aberrant chondroitin sulfate biosynthesis. These findings nominate PPARG, TCF12, CHST11, and CSGALNACT1 as candidate therapeutic targets and provide a conceptual framework for understanding how tubular-immune crosstalk shapes the renal inflammatory microenvironment in diabetic nephropathy.

## Conclusions

We identify a novel iPT-to-LAM intercellular communication axis in diabetic kidney disease, in which paracrine signals — including ANXA2 — from injured proximal tubular cells drive monocyte differentiation into a LAM state governed by PPARG- and TCF12-dependent transcriptional activation of chondroitin sulfate biosynthesis. These findings implicate macrophage-mediated glycosaminoglycan remodeling as a previously unrecognized effector mechanism in DKD and identify PPARG, TCF12, CHST11, and CSGALNACT1 as mechanistically grounded targets for future therapeutic investigation.

## Data Availability

The datasets presented in this study can be found in online repositories. The datasets are available under GEO accession numbers GSE211785 and GSE142025.

## Supplementary material

Supplementary Figure 1 Per-platform LAM frequency analysis. LAM proportions in SC-RNA-seq (Control: 0.04%, DKD: 0.40%) and SN-RNA-seq (Control: 27.1%, DKD: 50.8%) sub-datasets. Both platforms show consistent directional LAM expansion in DKD, supporting a robust disease-associated enrichment rather than a platform-specific or outlier-driven artifact.

Supplementary Figure 2 Cell-type-specific differential expression of CHST11 and CSGALNACT1 in DKD vs. Control across all 41 annotated cell populations. Macrophages exhibit the most significant upregulation of both genes in DKD (CHST11: log2FC = 3.09, p = 4.85×10^-^¹¹²; CSGALNACT1: log2FC = 2.83, p = 8.47×10^-59^), supporting LAM as the major cell population driving CS biosynthesis upregulation in the diabetic kidney microenvironment.

Supplementary Figure 3 CellChat-inferred incoming interaction strength toward LAM from all analyzed cell populations in DKD condition. iPT exhibits the highest incoming interaction strength (1.45×10^-^³), exceeding PT_S1 by approximately 5-fold (2.94×10^-4^) and all monocyte subpopulations by more than 10-fold, confirming iPT as the dominant paracrine signaling source toward LAM.
