# Supplementary material for: Injured proximal tubule-driven paracrine signaling promotes lipid-associated macrophage expansion and chondroitin sulfate biosynthesis via PPARG/TCF12 in diabetic kidney disease
Source: Front Immunol. 2026 Aug 20;17:1882611. doi: 10.3389/fimmu.2026.1882611 (PMC13538007; doi:10.3389/fimmu.2026.1882611)
Supplement: Supplementary file 1 [file DataSheet1.zip › Supplementary_Figure_1.pdf]

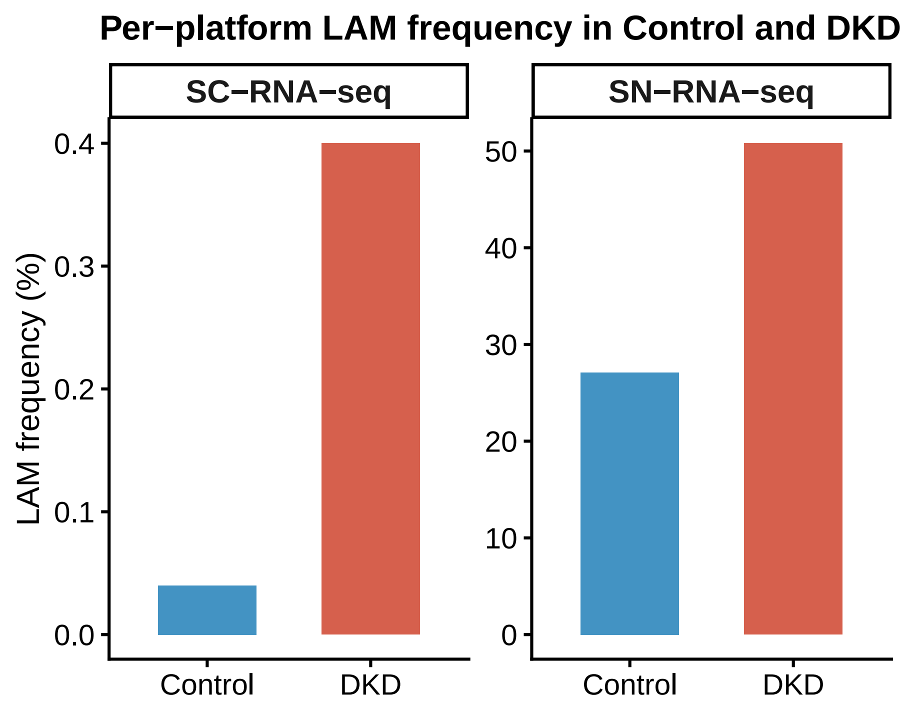

Per−platform LAM frequency in Control and DKD
SC−RNA−seq
SN−RNA−seq
LAM frequency (%)
0.0
0.1
0.2
0.3
0.4
0
10
20
30
40
50
Control
DKD
Control
DKD
