# Supplementary material for: Injured proximal tubule-driven paracrine signaling promotes lipid-associated macrophage expansion and chondroitin sulfate biosynthesis via PPARG/TCF12 in diabetic kidney disease
Source: Front Immunol. 2026 Aug 20;17:1882611. doi: 10.3389/fimmu.2026.1882611 (PMC13538007; doi:10.3389/fimmu.2026.1882611)
Supplement: Supplementary file 1 [file DataSheet1.zip › Supplementary_Figure_2.pdf]

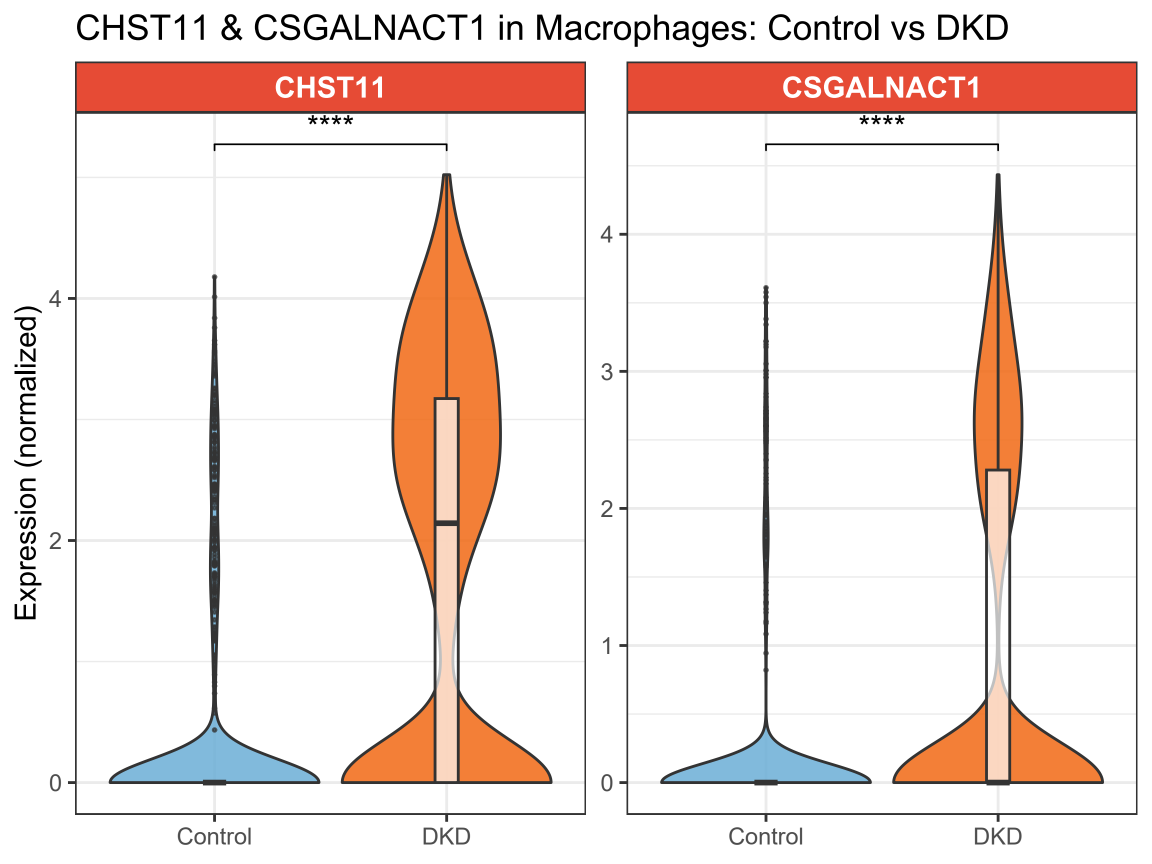

CHST11 & CSGALNACT1 in Macrophages: Control vs DKD
CHST11
CSGALNACT1
****
****
Expression (normalized)
0
2
4
0
1
2
3
4
Control
DKD
Control
DKD
