# Supplementary material for: Injured proximal tubule-driven paracrine signaling promotes lipid-associated macrophage expansion and chondroitin sulfate biosynthesis via PPARG/TCF12 in diabetic kidney disease
Source: Front Immunol. 2026 Aug 20;17:1882611. doi: 10.3389/fimmu.2026.1882611 (PMC13538007; doi:10.3389/fimmu.2026.1882611)
Supplement: Supplementary file 1 [file DataSheet1.zip › Supplementary_Figure_3.pdf]

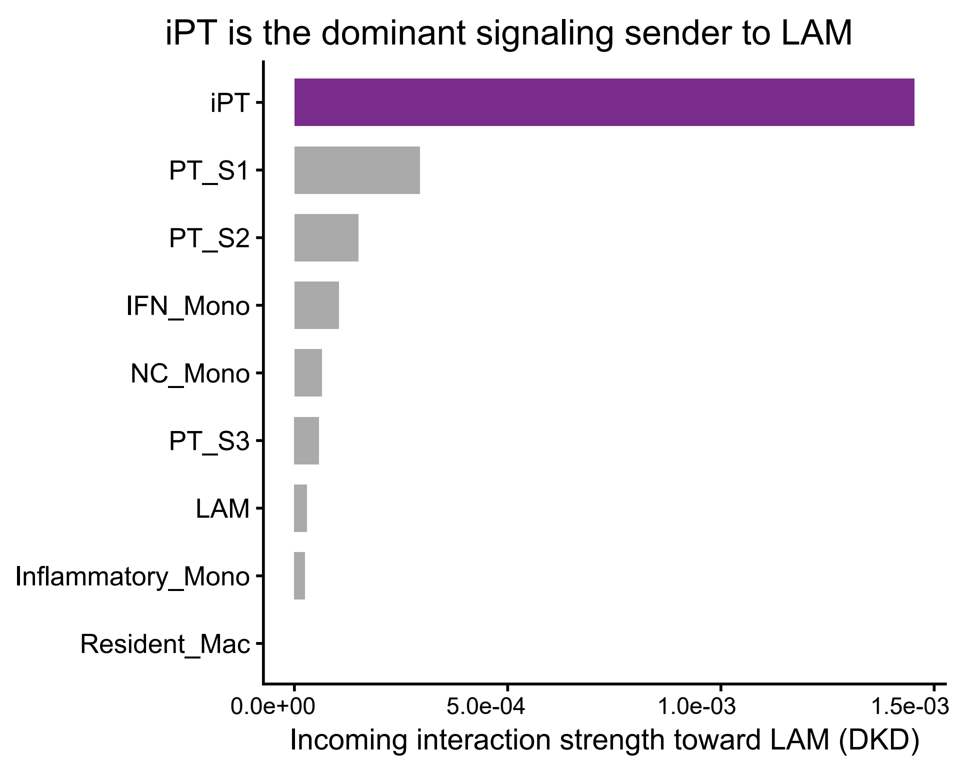

iPT is the dominant signaling sender to LAM
iPT
PT_S1
PT_S2
IFN_Mono
NC_Mono
PT_S3
LAM
Inflammatory_Mono
Resident_Mac
0.0e+00
5.0e-04
1.0e-03
1.5e-03
Incoming interaction strength toward LAM (DKD)
