# Supplementary material for: Injured proximal tubule-driven paracrine signaling promotes lipid-associated macrophage expansion and chondroitin sulfate biosynthesis via PPARG/TCF12 in diabetic kidney disease
Source: Front Immunol. 2026 Aug 20;17:1882611. doi: 10.3389/fimmu.2026.1882611 (PMC13538007; doi:10.3389/fimmu.2026.1882611)
Supplement: Supplementary file 1 [file DataSheet1.zip › Supplementary_Tables.docx]

# **Supplementary Tables**

**Article title:** Injured Proximal Tubule-driven Paracrine Signaling Promotes Lipid-associated Macrophage Expansion and Chondroitin Sulfate Biosynthesis via PPARG/TCF12 in Diabetic Kidney Disease

**Authors:** Bingshu Wu, Zihan Dou, Ming Cai, Siying Fei, Shuxuan Liu, Xiaobin Mei

**Corresponding authors:** Shuxuan Liu (332971271@qq.com); Xiaobin Mei (corresponding123@126.com)

## **Supplementary Table S1. qPCR Primer Sequences — HK-2 Cells**

| Gene | Direction | Sequence (5’→3’) | Source |
| --- | --- | --- | --- |
| ACTB | Forward | CATGTACGTTGCTATCCAGGC | PrimerBank ID: 4501885a1 (NM_001101) |
| ACTB | Reverse | CTCCTTAATGTCACGCACGAT | PrimerBank ID: 4501885a1 (NM_001101) |
| ANXA1 | Forward | TCTCCAGCAAAGCCAAGATG | PrimerBank (NM_000700) |
| ANXA1 | Reverse | GAAGTCCCGGTCACAAATGA | PrimerBank (NM_000700) |
| ANXA2 | Forward | ATGTCTACTGTCCACGAAATCCT | Ma et al., Mol Psychiatry 2019. doi:10.1038/s41380-019-0371-y |
| ANXA2 | Reverse | CGAAGTTGGTGTAGGGTTTGACT | Ma et al., Mol Psychiatry 2019. doi:10.1038/s41380-019-0371-y |
| THBS2 | Forward | GACACGCTGGATCTCACCTAC | PrimerBank ID: 40317627c1 (NM_003247) |
| THBS2 | Reverse | GAAGCTGTCTATGAGGTCGCA | PrimerBank ID: 40317627c1 (NM_003247) |
| CCN1 | Forward | CTGAGTGTATGCCATTCGG | Ju et al., Sci Rep 2020;10(1):3201 |
| CCN1 | Reverse | ACTGCTGTATCCCAATAAG | Ju et al., Sci Rep 2020;10(1):3201 |

## **Supplementary Table S2. qPCR Primer Sequences — CD14⁺ Monocyte-derived Macrophages**

| Gene | Direction | Sequence (5’→3’) | Source |
| --- | --- | --- | --- |
| HPRT1 | Forward | TGACACTGGCAAAACAATGCA | Valadan et al., Data in Brief 2015 (PMC4510554) |
| HPRT1 | Reverse | GGTCCTTTTCACCAGCAAGCT | Valadan et al., Data in Brief 2015 (PMC4510554) |
| PPARG | Forward | AGAAGCGGTGAACCACTGATATTC | Cifarelli et al., Genes Dev 2018. doi:10.1101/gad.312819.118 |
| PPARG | Reverse | AGAGGTCCACAGAGCTGATTCC | Cifarelli et al., Genes Dev 2018. doi:10.1101/gad.312819.118 |
| TCF12 | Forward | AGTTATCCATCTCCTAAGCCACC | PrimerBank ID: 46370079c1 (NM_207036) |
| TCF12 | Reverse | AAGAATTGTGGGTCCCATCTTG | PrimerBank ID: 46370079c1 (NM_207036) |
| FOXN3 | Forward | TCGTTGTGGTGCATAGACCC | PrimerBank ID: 146231999c2 (NM_001085471) |
| FOXN3 | Reverse | GTGGACCTGATGTGCTTTGATA | PrimerBank ID: 146231999c2 (NM_001085471) |
| CHST11 | Forward | AGAAATCAACCACCGCTTGAA | PrimerBank ID: 291219905c2 (NM_018413) |
| CHST11 | Reverse | GTAGGCGGACACTAGCCTC | PrimerBank ID: 291219905c2 (NM_018413) |
| CSGALNACT1 | Forward | GCTGTGCTATCTCTGTCCTGT | PrimerBank ID: 194473682c1 (NM_001130518) |
| CSGALNACT1 | Reverse | CTTCAGGCTGCTCACGTAGTT | PrimerBank ID: 194473682c1 (NM_001130518) |

**Supplementary Table S3. SCENIC-identified TF target genes not reaching significance in Reactome pathway enrichment analysis.**

The following 14 target genes regulated by PPARG, TCF12, and/or FOXN3 did not reach significance (p.adjust ≥ 0.05) in Reactome pathway enrichment analysis:

ATXN1, BNC2, CAMK1D, DISC1, ELMO1, EPB41L2, FAM53B, FMN1, KCNMA1, MGAT4A, PTPRJ, SFMBT2, SLCO2B1, SNTB1
